# Streamlined Vitamin D Metabolite Fingerprinting Analysis Using Isotope-Coded Multiplexing MS with Cost-Effective One-Pot Double Derivatization

**DOI:** 10.1021/acsomega.4c08675

**Published:** 2024-12-12

**Authors:** Pascal Schorr, Caroline S Stokes, Dietrich A Volmer

**Affiliations:** † Department of Chemistry, Humboldt Universität zu Berlin, Brook-Taylor-Str. 2, Berlin 12489, Germany; ‡ Thaer Institute, Humboldt Universität zu Berlin, Lentzeallee 75, Berlin 14195, Germany

## Abstract

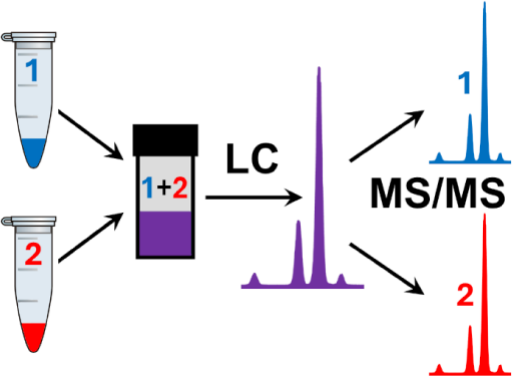

In this study, we extended a previously developed one-pot double derivatization reaction to establish the first routine isotope-coded multiplex derivatization for vitamin D and its metabolites for application in clinical environments, using commercial reagents, without the need for specialized reagents and advanced synthesis requirements. The original derivatization process consisted of using both a Cookson-type reagent and derivatization of hydroxyl groups. Initially, the analytes are derivatized by a Diels–Alder reaction using 4-phenyl-1,2,4-triazoline-3,5-dione (PTAD), followed by acetylation using acetic anhydride, catalyzed by 4-dimethylaminopyridine at room temperature. To enable sample multiplexing, we utilized acetic anhydride as well as the *d*_3_- isotopologue of acetic anhydride, generating *d*_3_- and *d*_6_-products of the investigated vitamin D_3_ metabolites. This approach not only allowed for the simultaneous measurement of two samples within a single LC-MS/MS run but also improved the LC separation of the important 25-hydroxyvitamin D_3_ epimers (3α-25(OH)D_3_ and 3β-25(OH)D_3_) on a conventional C-18 column, addressing a significant challenge in vitamin D analysis. Typically, the separation of these epimers after PTAD derivatization cannot be performed on C-18 columns, necessitating the use of pentafluorophenylpropyl (PFP) stationary phases. However, PFP columns are not as stable as C-18 in long-term use, wherein the acetylation of the C-3 hydroxyl group provided a solution by enhancing chromatographic selectivity and achieving the baseline separation of the metabolites 24,25(OH)_2_D_3_, 3α-25(OH)D_3_, 3β-25(OH)D_3_, and vitamin D_3_ using a C-18 column with methanol/water gradient elution. The described duplex derivatization was tested on 40 serum samples of patients with chronic liver diseases (CLD). Additionally, the method was evaluated in terms of linearity, accuracy, precision, and interferences between heavy and light tag samples using both commercial quality control samples and in-house quality control and calibration samples.

## 1 Introduction

During the COVID-19 pandemic, there was a sudden surge in the demand for vitamin D status testing, as several studies highlighted a link between vitamin D levels and the severity of COVID-19 infection.^1−3^ The role of the vitamin D sub-metabolome in many physiological and pathophysiological processes, however, had been investigated long before the pandemic. In addition to its crucial role in bone health by aiding the absorption of calcium and phosphorus from the intestine,^4,5^ vitamin D impacts various other health aspects. Vitamin D deficiency has been linked to numerous diseases, including depression,^6,7^ cardiovascular diseases,^4,8^ cancer,^4,9^ Alzheimer’s disease,^10,11^ and chronic liver disease.^12,13^

Vitamin D status is determined by measuring 25-hydroxyvitamin D levels in serum, but characterizing and quantifying other metabolites can provide a deeper understanding of the vitamin D sub-metabolome and its regulatory mechanisms. Clinically relevant metabolites include 24,25-dihydroxyvitamin D_3_ (24,25(OH)_2_D_3_), 1,25-dihydroxyvitamin D_3_ (1,25(OH)_2_D_3_), the epimers of 25-hydroxyvitamin D_3_ (3α-25(OH)D_3_ and 3β-25(OH)D_3_), and vitamin D_3_ itself. Determining the serum levels of 25(OH)D_3_ epimers separately is of clinical interest, as the biological function of 3α-25(OH)D_3_ is not yet fully understood. It is known that serum levels of 3α-25(OH)D_3_ are higher in infants, comprising up to 60% of total 25(OH)D_3_ serum levels, compared to ≤10% in adults,^14−18^ although levels can reach 24% in some cases.^14,16^

Liquid chromatography–mass spectrometry (LC-MS) is considered the “gold standard” method for determining vitamin D in blood serum.^19,20^ Due to the low serum concentrations of vitamin D metabolites, precolumn derivatization with Diels–Alder reagents is often used to enhance detection limits. Despite the need for analyzing samples from large sample cohorts in clinical studies, there is currently no commercial isotope-labeled derivatization reagent available for multiplexing and thus serving higher throughput purposes. Multiplexing refers to using derivatization reagents that differ in mass due to isotope labeling (=isotopologues) or modifications such as varying alkyl chain lengths,^21−23^ enabling the LC-MS analysis of multiple samples within the same run. While chemical modification of the derivatization reagent, such as the length of alkyl chains, allows for the simultaneous measurement of multiple samples, it also results in different retention times and varying matrix backgrounds. In contrast, isotope-coded derivatization reagents provide products exhibiting identical or nearly identical retention times.^22,24^ Isotope-coded multiplexing strategies have been successfully implemented in food safety,^25,26^ proteomics,^27−30^ and metabolomics applications,^31−34^ such as for the derivatization of carboxylic acids and fatty acids,^35−39^ usually requiring specialized derivatization reagents to be synthesized. Similarly, to date, multiplexing in the vitamin D analysis using isotope labeling has only been reported with in-house produced Cookson-type derivatization reagents.^22,23^ Since most bioanalytical or clinical laboratories performing clinical assays using LC-MS/MS lack the resources to synthesize such reagents, there is an urgent need for a commercially available isotope-coded derivatization reagent for multiplexing in vitamin D analysis. Netzel et al. implemented a multiplexing strategy for 25(OH)D_3_ and 25-hydroxyvitamin D_2_ (25(OH)D_2_) using five different Diels–Alder reagents.^21^ While this strategy worked for the determination of 25(OH)D_3_ and 25(OH)D_2_, no separation of different vitamin D_3_ metabolites was investigated. Furthermore, the retention time difference (0.2 min) compared to the retention time (0.5 min) was quite high, which underlines our point that a commercially available isotope-coded derivatization reagent for multiplexing in vitamin D analysis is needed.

In this study, we employed a one-pot double derivatization reaction,^40^ recently developed in our laboratory, to establish the first routine isotope-coded multiplex derivatization for vitamin D for application in clinical environments, using commercial reagents, without the need for specialized reagents and advanced in-house synthesis procedures. The original derivatization reaction (without isotope-coded reagents) was previously optimized, and the effects of the reagent concentration, addition of base, catalysts, and LC solvent additive methylamine were described.^40^ The sensitivity and increased separation power of the 25(OH)D_3_ epimers, as well as the stability of the reaction products of this one-pot double derivatization, compared to other derivatization reactions, are discussed elsewhere.^41,42^ The innovative approach combines a Cookson-type derivatization reagent, 4-phenyl-1,2,4-triazoline-3,5-dione (PTAD),^43^ with acetylation of the hydroxyl groups.^40^ Importantly, the isotope-coding is introduced in the acetylation step via deuterated and nondeuterated acetic anhydride, thus avoiding specialized and expensive reagents. PTAD specifically reacts with the diene moiety of vitamin D metabolites, forming stable products that enhance the detection sensitivity during analysis. The PTAD-vitamin D reaction products exhibit high protonation affinity and specific MS/MS behavior and shift the masses to higher *m*/*z* values with reduced isobaric noise. Unfortunately, the Diels–Alder reaction of PTAD with vitamin D metabolites produces two isomers (*R* and *S*), complicating the separation of 3α- and 3β-25(OH)D_3_, resulting in the need to separate four peaks. While challenging, this separation is feasible using pentafluorophenylpropyl (PFP) columns,^44^ although these columns tend to degrade faster than C-18 columns.^40,45^ To achieve separation on C-18 columns, we utilized a second derivatization step involving acetylation, catalyzed by 4-dimethylaminopyridine (DMAP).^40^ This second derivatization step also enabled multiplexing, as the derivatization reagent acetic anhydride is available in both regular (CH_3_CO)_2_O and deuterium-labeled forms of (CD_3_CO)_2_O.

## 2 Experimental Section

### 2.1 Chemicals and Materials

LC-MS grade methanol and acetonitrile were from VWR (Radnor, PA, USA); sodium chloride (>99.8%), anhydrous disodium hydrogen phosphate (>99%), potassium dihydrogen phosphate (>99%), bovine serum albumin (>98%, fatty acid-free), and acetic acid (100% p.a.) were from Carl Roth (Karlsruhe, Germany), formic acid (97%) was from Alfa Aesar (Karlsruhe, Germany), epimers of 25(OH)D_3_ were ordered as standard solutions, 3β-25(OH)D_3_ (100 μg·mL^–1^ in ethanol) and 3α-25(OH)D_3_ (50 μg·mL^–1^ in ethanol) were from Supelco (Bellefonte, PA, USA). All other metabolites were solids: 1,25(OH)_2_D_3_ from Cayman Chemical (Ann Arbor, MI, USA), *d*_6_-3α-25(OH)D_3_, *d*_6_-24,25(OH)_2_D_3_ from Endotherm (Saarbrücken, Germany), (24*R*)-24,25(OH)_2_D_3_ from Toronto Research Chemicals (Toronto, ON, Canada), and *d*_6_-3β-25(ΟΗ)D_3_ monohydrate from IsoSciences (Ambler, PA, USA). Lyophilized ClinChek Serum Controls for 25-OH vitamin D_2_/D_3_ (levels I and II) were obtained from Recipe (Munich, Germany) and reconstituted in water prior to analyses. Vitamin D_3_, *d*_3_-vitamin D_3_, *d*_6_-acetic anhydride, 4-dimethylaminopyridine (DMAP) (99%), 4-phenyl-1,2,4-triazoline-3,5-dione (PTAD) (97%), acetic anhydride (≥99%), pyridine (anhydrous, 99.8%), acetonitrile (anhydrous, 99.8%), methylamine solution in water (40%, *w/w*), and vitamin D-stripped human serum VD-DDC Mass Spect Gold serum were from Sigma-Aldrich (Steinheim, Germany). Organic-free water was generated using a Millipore (Bedford, MA, USA) Direct-Q8 purification system.

### 2.2 Reagents and Standard Solutions

PTAD reaction solution was freshly prepared as a 0.5 mg·mL^–1^ solution of PTAD in anhydrous acetonitrile containing 2% acetic acid and 2% *d*_3_-acetic acid for the heavy tag derivatization, respectively. Reaction solutions for acetylation were immediately prepared prior to the reaction from two parts of solution A (3 mg·mL^–1^ DMAP in pyridine) and one part of solution B (acetic anhydride) to give 2 mg·mL^–1^ DMAP in 2/1 (v/v) pyridine/acetic anhydride. For the heavy tag, we used *d*_6_-acetic anhydride as solution B and diluted with solution A to 2 mg·mL^–1^ DMAP in 2/1 (v/v) pyridine/*d*_6_-acetic anhydride. The isotope-labeled internal standard solution was prepared in acetonitrile with 30 ng·mL^–1^*d*_3_- vitamin D_3_, 75 ng·mL^–1^*d*_6_-3β-25(OH)D_3_, 45 ng·mL^–1^*d*_6_-3α-25(OH)D_3_, and 45 ng·mL^–1^*d*_6_-24,25(OH)_2_D_3_.

### 2.3 Sample Preparation

Serum samples (75 μL) were spiked with 10 μL of internal standard solution and then vortexed and incubated for 15 min, prior to adding 150 μL of acetonitrile for protein precipitation. Afterward the samples were vortexed for 30 s and centrifuged at 8,000*g* for 15 min. The supernatant was transferred to 1.5 mL reaction vials, and acetonitrile was removed under reduced pressure at 45 °C using an Eppendorf concentrator plus (Hamburg, Germany). For liquid–liquid extractions (LLE), 100 μL of water and 200 μL of ethyl acetate were added to the residue, vortexed for 30 s, and centrifuged for 5 min at 2,000*g*. Ethyl acetate was transferred to a fresh 1.5 mL reaction tube, and LLE was repeated with 200 μL of ethyl acetate. The organic phases were combined and dried under reduced pressure.

### 2.4 One-Pot Derivatization Reaction

For derivatization of the light tag, 25 μL of PTAD solution (0.5 mg·mL^–1^ in anhydrous acetonitrile/2% acetic acid) were added to the dried residue, vortexed, and left at 20 °C, protected from light. After reacting for 1 h, esterification was carried out by adding 25 μL of the freshly prepared esterification solution (2 mg·mL^–1^ DMAP in 2/1 (v/v) pyridine/acetic anhydride), followed by vortexing and reaction at 20 °C in the dark. After 1 h, the reaction was stopped by adding 50 μL of methanol, and samples were dried at 45 °C under reduced pressure. For the derivatization of the heavy tag, the derivatization steps were identical, except for using 0.5 mg·mL^–1^ PTAD in anhydrous acetonitrile +2% *d*_3_-acetic acid for the PTAD reaction and freshly prepared 2 mg·mL^–1^ DMAP in 2/1 (v/v) pyridine/*d*_6_-acetic anhydride for esterification. The dried derivatization products were dissolved in 50 μL of 90/10 methanol/water (v/v), and one light and one heavy tag samples were combined in one autosampler vial.

### 2.5 Serum Samples from CLD Patients

Serum samples of patients with chronic liver diseases (CLD) were provided by Saarland University Medical Center (Homburg, Germany). The study protocol was approved by the local research ethics committee. All participants provided written informed consent prior to their participation in the study. The research adhered to the ethical principles outlined in the Declaration of Helsinki. The serum samples were frozen at −80 °C until analysis. In this study, these samples were used to demonstrate the applicability of the new multiplexing derivatization scheme. Each sample was extracted and derivatized once and combined with the second sample as described above, and this combined sample extract was measured by LC-MS/MS in triplicate.

### 2.6 Method Evaluation and Calibration Curve

Vitamin D-depleted human serum VD-DDC Mass Spect Gold serum (specification: 25(OH)D_3_ < 1 ng·mL^–1^), adjusted to the required concentration of the investigated vitamin D_3_ metabolites, was used for in-house calibration and quality control (QC) samples. Concentrations of vitamin D_3_ metabolites in the vitamin D-depleted human serum were determined by standard addition. Calibration was achieved using seven-point calibration, and QC samples were prepared at three levels (QC_low_, QC_med_, QC_high_). In addition to in-house QC samples, commercially available, certified QC samples for 3β-25(OH)D_3_, ClinChek Serum Control for 25-OH vitamin D2/D3 (levels I and II), were processed in triplicate. Phosphate-buffered saline (PBS) at pH 7.4 was prepared under the FDA guideline BAM R59, by dissolving 7.650 g of sodium chloride, 0.724 g of anhydrous disodium hydrogen phosphate, and 0.210 g of potassium dihydrogen phosphate in 1 L of distilled water and adjusting pH to 7.4. Bovine serum albumin (BSA) solution was prepared by dissolving 4.2 g of BSA in 100 mL of PBS. Each calibration level was extracted and derivatized in triplicate, and each sample was measured by LC-MS/MS in triplicate. Sciex (Concord, ON, Canada) MultiQuant 3.0.3 software with 1/*x* weighting was used to create calibration curves and to evaluate data of patient and QC samples. Accuracy and precision were expressed as relative error (RE) and relative standard deviation (RSD) as follows: RE = 100 × (measured- theoretical conc.) × (theoretical conc.)^−1^ and RSD = 100 × (standard deviation) × (average measured conc.)^−1^.^46^ To check for possible MRM crossover interaction of two samples with high variations in their vitamin D metabolite levels, QC samples at high and low levels were derivatized with the light and the heavy tags, respectively; QC_low_ was then mixed with QC_high_. Three replicas of each combination A: QC_low_ (light tag) with QC_high_ (heavy tag) and B: QC_low_ (heavy tag) with QC_high_ (light tag) were analyzed in triplicate, and RE was determined to evaluate possible MRM crossover interactions.

### 2.7 LC-MS/MS

LC-MS/MS of derivatized duplex samples was performed with an Agilent (Wilmington, DE, USA) 1290 Infinity II UHPLC system coupled to a Sciex QTRAP 6500+ quadrupole-linear ion trap (QqLIT) MS. 5 μL of each sample were injected in triplicate onto a Phenomenex (Torrance, CA, USA) Kinetex XB C-18 column (100 × 2.1 mm, 2.6 μm, 100 Å) maintained at 35 °C. Separations were achieved using gradient elution at 0.4 mL min^–1^ with (A) 2.5 mM methylamine in water +0.1% formic acid and (B) 2.5 mM methylamine in methanol +0.1% formic acid as mobile phases. The gradient elution program started at 75% B, remained for 3 min at 75% B, and increased within 3.5 min to 81.5% B. The gradient further increased to 96.3% B in 4 min and afterward increased to 100% B within 0.1 min, was held there for 1.9 min, and decreased to 75% in 0.1 min, followed by equilibration under starting conditions for 1.4 min. The QTRAP instrument was operated in positive ion mode by using electrospray ionization (ESI). The ion source parameters were: ESI voltage: 5500 V; curtain gas: 35 psi; source temperature: 350 °C; sprayer gas (gas 1), 30 psi; heater gas (gas 2), 38 psi. For MRM, the collision gas pressure was set to medium, and MS parameters were optimized for each analyte, separately (Table S1).

## 3 Results and Discussion

We recently described a one-pot double derivatization method for vitamin D metabolites.^40^ This method employs a Diels–Alder reaction of vitamin D metabolites with PTAD to increase the detection sensitivity, followed by an esterification step with acetic anhydride to improve the separation of 3α- and 3β-25(OH)D_3_ epimers. The objective of that method was to facilitate quantitative profiling of key vitamin D metabolites, including the critical 25(OH)D_3_ epimers. In the present study, we describe a new cost-effective multiplex assay leveraging the previous PTAD enhancements and epimer separation capabilities. Instead of the acetic anhydride derivatization step, we incorporated isotope-labeled acetic anhydride (CD_3_CO)_2_O (heavy tag) into the protocol. This modification allows for the simultaneous analysis of two samples (one with a heavy and one with a light tag) in a single LC-MS/MS run with samples distinguished by their MRM transitions.

**Figure 1:**
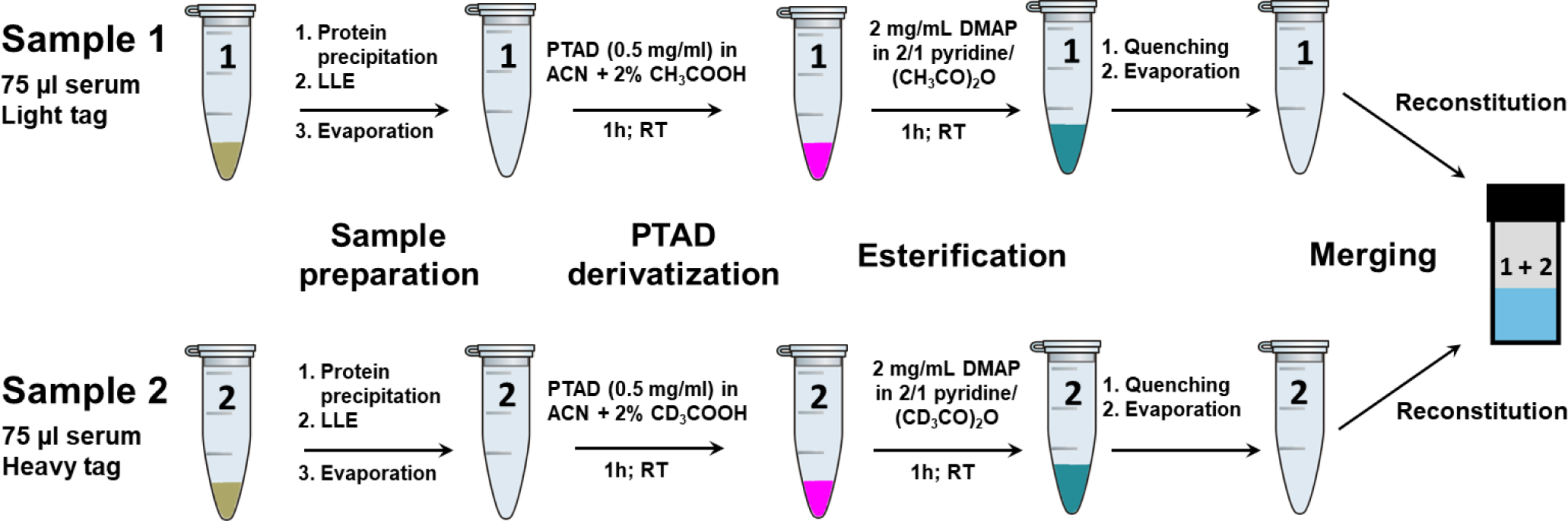
Derivatization sequence for light- and heavy-tagged samples.

**Figure 2:**
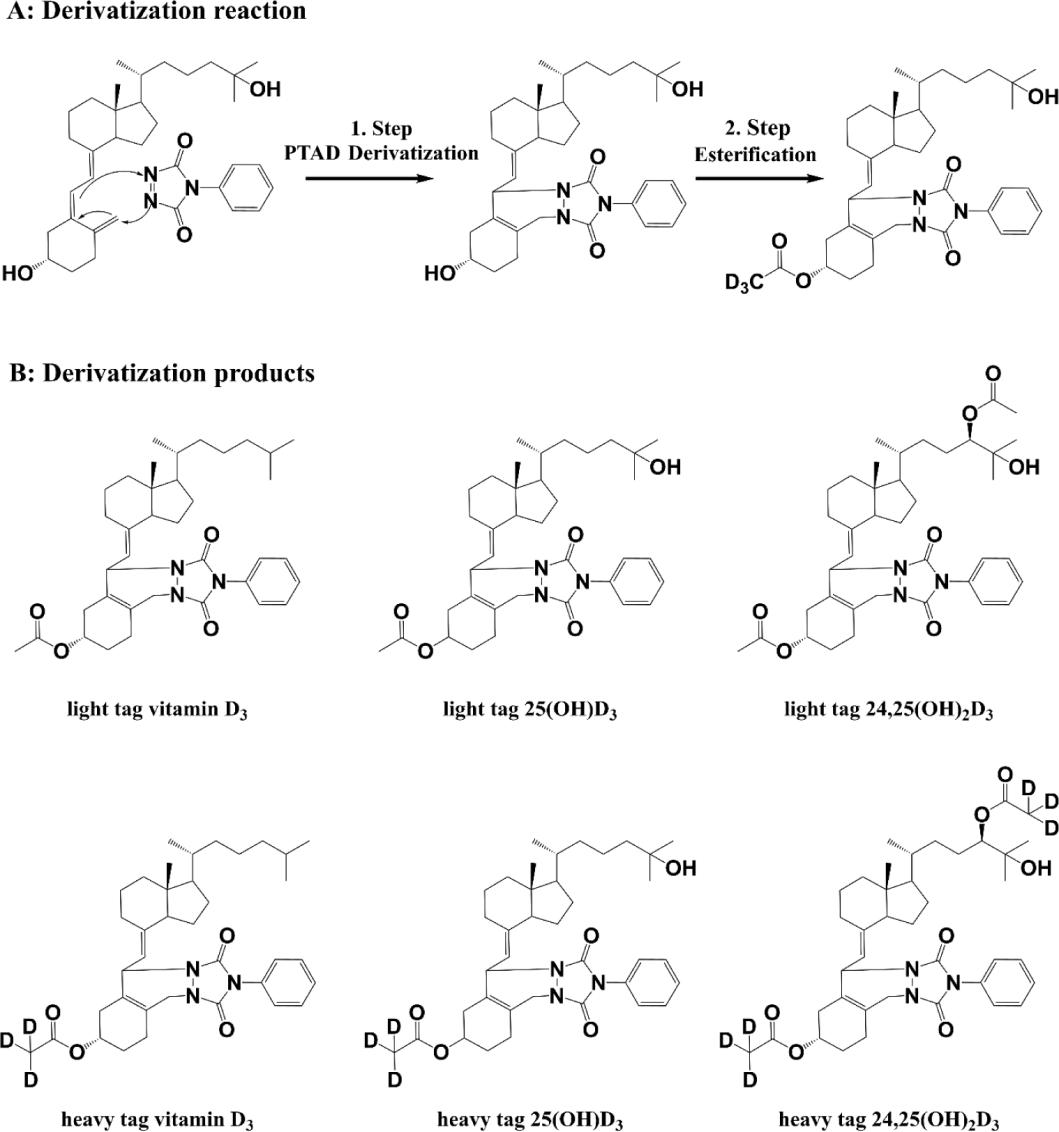
A: Reaction mechanism of the one-pot double derivatization exemplified for the heavy tag of 3β-25(OH)D_3_ using PTAD derivatization and DMAP-catalyzed acetylation; B: light and heavy tag derivatization products of 24,25(OH)_2_D_3_, 25(OH)D_3_,and vitamin D_3_.

The derivatization and sample handling procedures are summarized in Figure 1. The derivatization reaction of the one-pot double derivatization for the heavy tag sample of 25(OH)D_3_, along with the light and heavy tag derivatization products for 24,25(OH)_2_D_3_, 25(OH)D_3_, and vitamin D_3_, is illustrated in Figure 2. The detailed reaction mechanism has been described previously.^40^

We carefully evaluated this new duplexing method in the LC-MS/MS analysis of vitamin D_3_ and selected metabolites. Calibration checks were performed, by simulating worst-case scenarios of low-level samples mixed with high-level samples, to ensure no MRM crossover interactions between light and heavy tag samples or with their isotope-labeled internal standards occur. Additionally, we used certified commercial QC serum and in-house QC serum samples with varying concentrations of vitamin D_3_ metabolites. Furthermore, this derivatization protocol and the LC-MS/MS method were applied to analyze serum samples from a cohort of CLD patients.

### 3.1 Duplexing the Derivatization Reaction

The first step in the derivatization protocol involves reacting the vitamin D metabolites with PTAD in dry acetonitrile containing 2% acetic acid or deuterium-labeled acetic acid. The addition of acetic acid enhances the reaction yield.^40^ In this step, the diene moiety of the vitamin D metabolites undergoes a Diels–Alder reaction with the azo group of PTAD, forming a six-membered ring and resulting in two isomers (6*R*- and 6*S*-) of each metabolite.

In the second step, esterification with acetic anhydride, catalyzed by 4-dimethylaminopyridine (DMAP), is performed. All hydroxyl groups, except at the C-25 position, are esterified (Figure 2). DMAP activates acetic anhydride by forming a reactive acetylpyridinium ion intermediate, which then reacts with the hydroxyl groups of the vitamin D metabolites to form ester products. DMAP binds the proton from the released acetic acid and is regenerated by pyridine through deprotonation. Unlabeled acetic anhydride is used for the light tag sample, while deuterium-labeled acetic anhydride is used for the heavy tagged sample. After derivatization, both samples are combined in one vial (Figure 1).

### 3.2 Liquid Chromatography Separation

In addition to enabling the simultaneous measurement of two samples, the described derivatization procedure achieved the baseline separation of the two critical epimers of 25(OH)D_3_ on a conventional C-18 column. Initially, the primary focus of the double derivatization was this particular epimer separation.^40^ Without the esterification step, the separation of the epimers after PTAD derivatization was not possible, and this step was crucial for detecting low levels of 3α-25(OH)D_3_, 24,25(OH)2D_3_, and vitamin D_3_.

**Figure 3:**
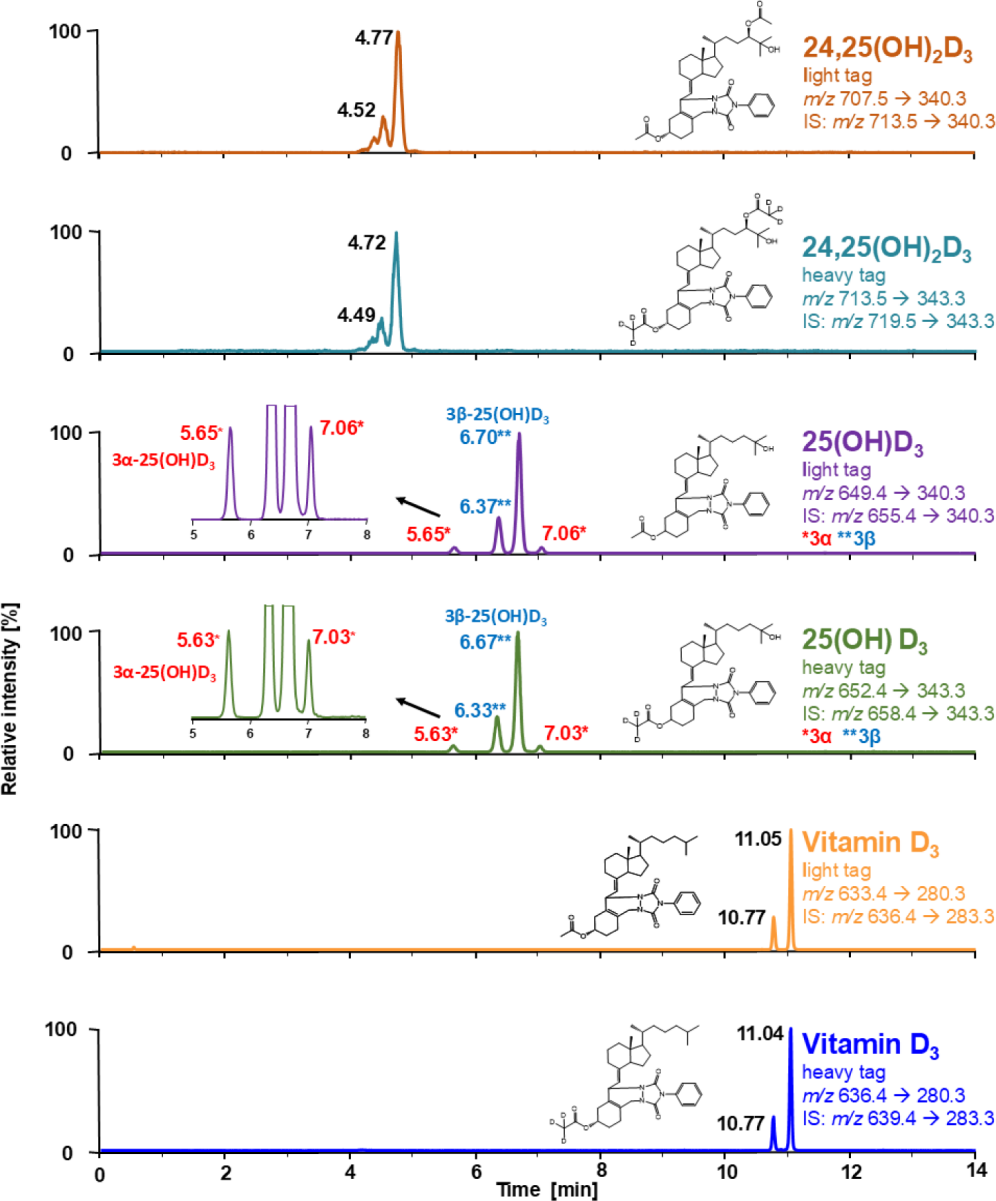
LC-MS/MS MRM chromatograms of serum samples with the light and heavy-tagged vitamin D_3_ metabolites.

Importantly, the use of isotope-labeled tags and the addition of methylamine as an additive did not affect the retention times or separation efficiency. As illustrated in Figure 3, the retention times of all four metabolites were nearly identical between the light and heavy tagged samples, with retention time differences below 0.05 min, which are on the same order as MRM transition time windows. Methylamine was added to enhance sensitivity and improve LC-MS/MS chromatograms, as previously described.^40,43^

### 3.3 Evaluation of Calibration and QC Samples

The calibration curve and in-house quality control samples were prepared by using vitamin D-depleted serum. This serum contained residual vitamin D_3_ at a concentration of 0.70 ng/mL, determined by standard addition. To achieve a lower calibration level (L1) for vitamin D_3_, the vitamin D-depleted serum was diluted with a BSA solution to reach 0.35 ng/mL. For other metabolites, the serum was spiked to the desired calibration levels without further dilution.

The calibration range and determination coefficient (*R*^2^) are provided in Table 1, while the accuracy and precision of the individual levels are detailed in Table S2. The calibration for the four metabolites exhibited excellent linearity with *R*^2^ > 0.999. The light and heavy tags showed no differences in linearity.

**Table 1:** Precision and Accuracy of the Analysis of Quality Control Samples Expressed as Relative error (RE) and Relative Standard Deviation (RSD, *n* = 3) in %^a^

		24,25(OH)_2_D_3_				3α-25(OH)D_3_				3β-25(OH)D_3_				Vitamin D_3_			
Quality control samples		Light tag		Heavy tag		Light tag		Heavy tag		Light tag		Heavy tag		Light tag		Heavy tag	
Light tag	Heavy tag	RE [%]	RSD [%]	RE [%]	RSD [%]	RE [%]	RSD [%]	RE [%]	RSD [%]	RE [%]	RSD [%]	RE [%]	RSD [%]	RE [%]	RSD [%]	RE [%]	RSD [%]
QC low	QC low	8.4	2.9	12.2	2.9	0.7	2.4	4.2	1.8	0.2	3.7	4.7	3.7	0.2	5.1	1.0	3.4
QC med	QC med	1.9	4.0	11.7	2.4	3.8	4.2	7.3	3.5	4.2	3.7	5.5	2.0	8.6	3.5	1.3	3.7
QC high	QC high	2.7	1.0	4.6	2.7	2.8	2.2	3.2	1.1	2.4	1.2	0.8	1.8	1.6	1.2	2.3	2.2
QC low	QC high	10.0	2.7	3.5	1.4	1.1	3.0	2.3	1.7	1.4	2.6	1.9	2.1	0.7	1.8	3.6	2.5
QC high	QC low	1.4	1.9	13.6	0.9	1.5	2.4	4.0	0.6	2.5	2.3	2.6	2.1	1.4	1.6	11.0	2.7
Recipe Lv. I^b^	Recipe Lv. I^b^	-	-	-	-	-	-	-	-	2.6	0.6	1.7	1.6	-	-	-	-
Recipe Lv. II^b^	Recipe Lv. II^b^	-	-	-	-	-	-	-	-	1.4	0.3	1.9	2.4	-	-	-	-
Determination coefficient *R*^2^		0.9998		0.9998		0.9997		0.9998		0.9995		0.9994		0.9996		0.9998	
Calibration Range		0.1–25 ng mL^–1^				0.1–25 ng mL^–1^				1–100 ng mL^–1^				0.35–69.7 ng mL^–1^			
Conc. QC low		0.5 ng mL^–1^				0.5 ng mL^–1^				4 ng mL^–1^				1.2 ng mL^–1^			
Conc. QC med		2.0 ng mL^–1^				2.0 ng mL^–1^				25 ng mL^–1^				15.7 ng mL^–1^			
Conc. QC high		20 ng mL^–1^				20 ng mL^–1^				85 ng mL^–1^				50.7 ng mL^–1^			
Recipe Lv. I^b^		-				-				14.9 ng mL^–1^				-			
Recipe Lv. II^b^		-				-				42.0 ng mL^–1^				-			

a Calibration range and correlation coefficient *R*^2^ of the calibration curves

b Recipe ClinCheck Serum control Level I and II reference standards were only available for 3β-25(OH)D_3_ and 3β-25(OH)D_2_ interferences with the internal standard.

Values for 3β-25(OH)D_3_ measured in commercially available quality control serum were consistent with the stated concentrations of 14.9 and 42.0 ng/mL. The accuracy, expressed as the relative error (RE), was ≤2.6%, and precision, expressed as the relative standard deviation (RSD), was ≤2.4%. Due to the unavailability of commercial quality control serum for 24,25(OH)2D_3_, 3α-25(OH)D_3_, and vitamin D_3_, in-house QC samples were used. For the sample with the heavy tag, RE was ≤13.6% and RSD was ≤3.7%. For the light tag, RE was ≤10.0% and RSD ≤ 5.1%. All calibration and QC samples met the acceptance criteria for RE and RSD of ≤15%, as specified by the FDA Bioanalytical Guidelines.^47^

The recovery of the sample preparation, intra- and interday precision, accuracy, and stability of the one-pot double derivatization method have been previously demonstrated.^40,42^

### 3.4 Cross-Interferences between Heavy and Light Tag Samples

To evaluate potential interferences between the mixed samples, we investigated a worst-case scenario: mixing a high-concentration-level sample with a low-level sample. In these experiments, we used in-house QC samples at both low and high concentration levels. The results are summarized in Table 1. When a QC_low_ sample (light tag) was mixed with a QC_high_ sample (heavy tag), the accuracy and precision were comparable to the mixture of two samples with identical analytical concentrations

The QC_low_ sample (light tag) exhibited the lowest accuracy for 24,25(OH)_2_D_3_, with a relative error of 10.0% and a relative standard deviation of 2.7%. The other metabolites demonstrated excellent accuracy, with RE ≤ 1.4% and RSD ≤ 3.0%. There was no significant difference in the measured concentration of 24,25(OH)_2_D_3_ compared to a mixture of two QC_low_ samples, which had an RE of 8.4% and an RSD of 2.9%. The QC_high_ sample (heavy tag) showed good agreement with the theoretical values for all four analytes, with RE ≤ 3.6% and RSD ≤ 2.5%.

For the reverse scenario, where a QC_low_ sample (heavy tag) was mixed with a QC_high_ sample (light tag), the QC low sample had the highest RE for 24,25(OH)_2_D_3_ at 13.6% and an RSD of 0.9%. This was comparable to mixing two QC_low_ samples, which yielded an RE of 12.2% and an RSD of 2.9%, thus showing that accuracy and precision are comparable. For vitamin D_3_, the RE for the QC_low_ sample (heavy tag) was higher at 11.0%, compared to an RE of 1.0% for the QC_low_ sample mixture. However, this experiment aimed to demonstrate a worst-case scenario, and the measured concentrations remained within the FDA Bioanalytical Method Validation Guidelines’ acceptance criteria, which allow for 15% RE and RSD.^47^ The QC high sample (light tag) showed good agreement with the theoretical values for all four analytes, with RE ≤ 2.5% and RSD ≤ 2.4%

In this study, we utilized deuterium-labeled acetic anhydride to develop a multiplexing LC-MS/MS method, creating *d*_3_- and *d*_6_-derivatization products of the analyzed vitamin D_3_ metabolites. Given that the internal standards are also *d*_3_- and *d*_6_-labeled vitamin D_3_ metabolites, the existence of potential interferences needed to be considered. However, for 3α- and 3β-25(OH)D_3_, there was no interference because we used *d*_6_-25(OH)D_3_ as the internal standard, and the derivatization generated a *d*_3_-derivative of 25(OH)D_3_ for both epimers.

**Figure 4:**
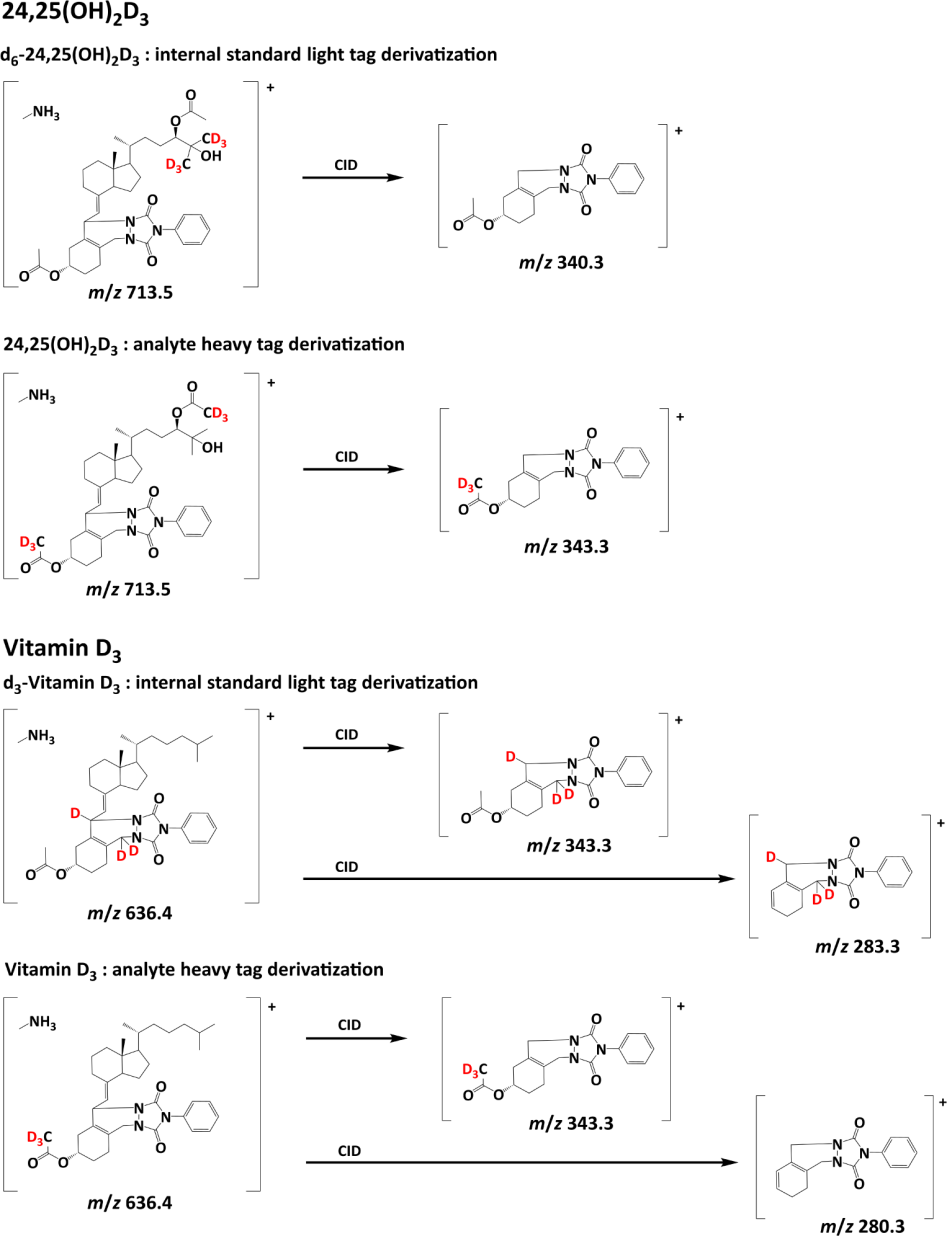
Fragmentation pattern and MRM ion selection for light/heavy tag derivatization products and isotope internal standards.

For 24,25(OH)_2_D_3_, we employed *d*_6_-24,25(OH)_2_D_3_ as the internal standard. The derivatization resulted in two esterifications with *d*_3_-acetic acid, forming a *d*_6_-product of 24,25(OH)_2_D_3_ in the heavy tag sample. As depicted in Figure 4, the analyte of the heavy tag sample therefore has a mass identical to that of the internal standard of the light tag sample, sharing the same precursor ion. However, due to the different deuterium-labeled positions, they can be separated after collision-induced dissociation (CID) of the C-6/7 bond in the vitamin D_3_ structure. This characteristic A-ring fragmentation pattern is the preferred transition for MRM of PTAD-derivatized vitamin D metabolites, as it yields the highest intensities.^48^

For vitamin D_3_ analysis, we used *d*_3_-vitamin D_3_ as the internal standard. Consequently, with the *d*_3_-acetylation of the heavy tag derivatization reaction, isobaric interference between the internal standard of the light tag and the analyte of the heavy tag is theoretically possible. As shown in Figure 4, the acetylation of the hydroxyl group at C-3 and the deuterium label at C-6 and C-19 on the internal standard precludes the use of A-ring fragmentation for MRM. Instead, further fragmentation with a neutral loss of acetic acid provides a suitable MRM transition for quantitative analysis. However, the LOQ using this MRM transition was lower compared to using A-ring fragmentation, mainly due to higher background noise in the MRM transition for the heavy tag. This issue can be resolved by using *d*_6_-vitamin D_3_ as an internal standard, allowing for the use of A-ring fragmentation for MRM in quantitative analysis. Unfortunately, *d*_6_-vitamin D_3_ was unavailable at the time of this study but is expected to be commercially available in the future, which will permit measurements of lower vitamin D_3_ levels while employing this derivatization assay for multiplexing.

### 3.5 Application to Serum Samples

To demonstrate the application of the developed assay, levels of 24,25(OH)_2_D_3_, 3α-25(OH)D_3_, 3β-25(OH)D_3_, and vitamin D_3_ were measured in 40 serum samples from patients with chronic liver diseases (CLD) (Table 2). We successfully quantified 3α- and 3β-25(OH)D_3_ in all samples, with a low standard deviation between measurements (RSD of ≤8.4% for 3α-25(OH)D_3_ and ≤2.8% for 3β-25(OH)D_3_).

**Table 2:** Measured Concentration Levels of Vitamin D Metabolites in Patient Samples^a^

Sample	Tag	24,25(OH)2D_3__;_ meas. conc (±SD, *n* = 3) [ng mL^–1^]	3α-25(OH)D_3__;_ meas. conc (±SD, *n* = 3) [ng mL^–1^]	3β-25(OH)D_3__;_ meas. conc (±SD, *n* = 3) [ng mL^–1^]	Vitamin D_3__;_ meas. conc (±SD, *n* = 3) [ng mL^–1^]
1	L	1.64 (±0.04)	1.06 (±0.03)	19.4 (±0.1)	0.53 (±0.04)
2	L	1.94 (±0.05)	1.62 (±0.06)	21.3 (±0.2)	0.90 (±0.04)
3	L	0.82 (±0.04)	0.86 (±0.03)	13.3 (±0.1)	<LLOQ
4	L	0.96 (±0.04)	1.07 (±0.07)	17.8 (±0.2)	0.49 (±0.01)
5	L	0.71 (±0.02)	0.75 (±0.01)	14.2 (±0.0)	0.38 (±0.02)
6	L	0.40 (±0.00)	0.56 (±0.02)	10.5 (±0.1)	<LLOQ
7	L	1.62 (±0.05)	2.39 (±0.14)	20.1 (±0.2)	2.16 (±0.08)
8	L	1.63 (±0.06)	1.55 (±0.1)	25.7 (±0.2)	0.70 (±0.03)
9	L	2.96 (±0.07)	2.19 (±0.05)	25.4 (±0.3)	1.24 (±0.01)
10	L	0.14 (±0.01)	0.35 (±0.03)	6.2 (±0.1)	<LLOQ
11	L	1.00 (±0.04)	1.26 (±0.09)	14.4 (±0.2)	0.36 (±0.01)
12	L	0.34 (±0.01)	1.29 (±0.01)	29.8 (±0.3)	0.36 (±0.00)
13	L	0.39 (±0.01)	0.31 (±0.01)	9.1 (±0.1)	<LLOQ
14	L	1.04 (±0.04)	1.61 (±0.05)	18.6 (±0.1)	<LLOQ
15	L	0.93 (±0.02)	0.85 (±0.04)	15.1 (±0.4)	<LLOQ
16	L	0.31 (±0.01)	0.98 (±0.04)	8.7 (±0.2)	0.42 (±0.01)
17	L	0.25 (±0.01)	0.67 (±0.02)	4.9 (±0.1)	<LLOQ
18	L	2.73 (±0.11)	1.76 (±0.04)	37.3 (±0.4)	3.93 (±0.12)
19	L	1.00 (±0.03)	1.32 (±0.02)	19.4 (±0.3)	0.62 (±0.02)
20	L	1.57 (±0.03)	1.95 (±0.03)	13.2 (±0.2)	<LLOQ
21	H	0.25 (±0.02)	1.14 (±0.01)	18.7 (±0.1)	15.49 (±0.15)
22	H	0.51 (±0.01)	1.21 (±0.09)	16.8 (±0.1)	<LLOQ
23	H	0.20 (±0.02)	0.23 (±0.01)	6.7 (±0.1)	<LLOQ
24	H	4.19 (±0.06)	2.45 (±0.10)	23.7 (±0.1)	3.35 (±0.05)
25	H	<LLOQ	0.31 (±0.00)	2.2 (±0.1)	<LLOQ
26	H	<LLOQ	0.10 (±0.01)	2.4 (±0.0)	0.42 (±0.01)
27	H	0.63 (±0.02)	0.90 (±0.03)	24.2 (±0.4)	12.01 (±0.03)
28	H	0.86 (±0.03)	1.98 (±0.1)	21.6 (±0.3)	4.34 (±0.11)
29	H	2.06 (±0.06)	2.09 (±0.02)	26.7 (±0.2)	2.50 (±0.07)
30	H	0.80 (±0.02)	1.05 (±0.04)	18.7 (±0.0)	2.08 (±0.1)
31	H	1.09 (±0.01)	0.71 (±0.01)	11.8 (±0.1)	<LLOQ
32	H	0.40 (±0.02)	0.54 (±0.02)	7.6 (±0.1)	<LLOQ
33	H	0.31 (±0.02)	0.21 (±0.00)	4.5 (±0.1)	<LLOQ
34	H	0.85 (±0.01)	1.73 (±0.04)	18.2 (±0.2)	16.54 (±0.22)
35	H	0.77 (±0.03)	0.58 (±0.02)	15.6 (±0.4)	0.88 (±0.08)
36	H	1.68 (±0.04)	5.71 (±0.09)	43.0 (±1.2)	18.01 (±0.19)
37	H	1.20 (±0.03)	2.05 (±0.07)	16.5 (±0.3)	4.55 (±0.06)
38	H	3.18 (±0.09)	0.99 (±0.02)	24.4 (±0.3)	2.4 (±0.2)
39	H	0.33 (±0.01)	0.56 (±0.02)	6.3 (±0.2)	<LLOQ
40	H	4.11 (±0.06)	4.02 (±0.02)	31.2 (±0.2)	6.56 (±0.16)

a L: light tag sample; H: heavy tag sample

Serum levels of 3α-25(OH)D_3_ ranged from 0.10 ng mL^–1^ to 5.71 ng mL^–1^ and levels of 3β-25(OH)D_3_ ranged from 2.2 ng mL^–1^ to 43.0 ng mL^–1^. In 38 out of 40 serum samples, 24,25(OH)_2_D_3_ was quantifiable with levels between 0.14 and 4.19 ng mL^–1^; the other two samples were below the LOQ of 0.10 ng mL^–1^. For vitamin D_3_, 15 samples were below the LOQ of 0.35 ng mL^–1^. The serum concentration of vitamin D_3_ in the remaining 25 serum samples ranged from 0.36 to 18.01 ng mL^–1^. The relative standard deviation between measurements was ≤9.8% for 24,25(OH)_2_D_3_ and ≤9.4% for vitamin D_3_.

As noted in the previous section, to measure lower levels of vitamin D_3_, we recommend using *d*_6_-vitamin D_3_ instead of *d*_3_-vitamin D_3_ as the internal standard. Using *d*_6_-vitamin D_3_ allows for a more sensitive MRM transition without isobaric interference between the internal standard and analyte in the light and heavy tag samples.

## 4 Conclusions

In this study, we employed unlabeled and deuterium-labeled acetic acid in a one-pot double derivatization reaction, generating both light and heavy tagged vitamin D_3_ metabolites. This derivatization enabled us to establish the first isotope-coded multiplexing LC-MS/MS method for vitamin D_3_ fingerprinting using inexpensive, commercially available reagents without the need for specialized in-house syntheses. In addition to allowing the measurement of two samples in a single LC-MS/MS run, this method also facilitated the separation of the 3α- and 3β-25(OH)D_3_ epimers on conventional C-18 columns.

The 3α- and 3β-25(OH)D_3_ metabolites were readily quantifiable in all investigated CLD patient serum samples, while 24,25(OH)_2_D_3_ was determined in 38 out of 40 samples; vitamin D_3_ was determined in 25 out of 40 samples. The lower quantification rate for vitamin D_3_ was due to the use of *d*_3_-vitamin D_3_ as an internal standard, which caused interferences. The chosen alternate MRM transition exhibited elevated background noise for the heavy tag sample, resulting in a higher LOQ. Using *d*_6_-vitamin D_3_ as an internal standard in the future will avoid this isobaric interference and is recommended for analyzing low levels of vitamin D_3_.

We also studied potential crossover interferences between two samples with low and high concentrations, finding them to be acceptable according to the FDA guidelines with RE and RSD < 15%.

This derivatization assay could be readily extended to analyze more than two samples by using additional anhydrides, such as propionic anhydride, for which a deuterated isotopologue is commercially available (the additional −CH_2_ group is not expected to significantly affect retention times). With this combination, duplexing would extend to the simultaneous analysis of four samples. Further expansion is feasible by using fluorinated acetic acids (mono-, di-, and trifluoroacetic acid).

Finally, in addition to the multiplexing capabilities, the differential nature of the isotope-coded label described here also permits relative quantitative analyses. This approach allows for the simultaneous analysis of two physiological states or the monitoring of changes in an individual’s metabolite profile over time. For instance, metabolic changes in vitamin D metabolites can be tracked throughout medical treatment or nutritional intervention. At the initial time point (*t* = 0), before treatment/intervention starts, the sample can be tagged with the more expensive heavy isotope label, while subsequent time point samples are each tagged with the less expensive nonlabeled tag, each then mixed with the *t* = 0 sample. The measured *d*_0_/*d*_3_ ratios, along with the variations of these ratios, provide accurate information on the up- and down-regulation of the vitamin D metabolites over the course of the study. Unlike conventional analyses, which are affected by interassay variability, the assay developed in this study completely eliminates such variations, ensuring accurate comparability of an individual’s concentration levels.

## Supporting Information Available

Optimized MS parameters for MRM experiments; concentration levels and precision and accuracy of calibration samples expressed as relative error (RE) and relative standard deviation (PDF)
