# Supplementary material for: Streamlined Vitamin D Metabolite Fingerprinting Analysis Using Isotope-Coded Multiplexing MS with Cost-Effective One-Pot Double Derivatization
Source: ACS Omega. 2024 Dec 12;9(51):50660–70. doi: 10.1021/acsomega.4c08675 (PMC11684529; doi:10.1021/acsomega.4c08675)
Supplement: Supplementary file 1 — ao4c08675_si_001.pdf [file ao4c08675_si_001.pdf]

Supporting Information

Streamlined vitamin D metabolite fingerprinting analysis using isotope-coded multiplexing MS with cost-effective one-pot double derivatization

Pascal Schorr,[1] Caroline S. Stokes,[2] Dietrich A. Volmer[1]*

[1]Department of Chemistry, Humboldt Universität zu Berlin, Brook-Taylor-Str. 2, 12489 Berlin, Germany

[2]Thaer Institute, Humboldt Universität zu Berlin, Lentzeallee 75, 14195 Berlin, Germany

*Corresponding author:
Prof. Dr. Dietrich A. Volmer
Department of Chemistry
Humboldt-Universität zu Berlin
Brook-Taylor-Str. 2
12489 Berlin, Germany
Tel: +49 30 2093 7588
Email: dietrich.volmer@hu-berlin.de

Table S1: Optimized MS parameters for MRM experiments.

Analyte / IS	Tag	Precursor ion *m/z*	Product ion *m/z*	Collision energy	Dwell time [ms]	Declustering potential	Entrance potential	Collison cell exit potential
24,25$(OH)_2D_3$	Light	707.5	340.3	30	40	45	11	15
d_6-24,25$(OH)_2D_3$ (IS)	Light	713.5	340.3	30	40	45	11	15
3α-25$(OH)D_3$	Light	649.4	340.3	28	40	45	11	15
d_6-3α-25$(OH)D_3$ (IS)	Light	655.4	340.3	28	40	45	11	15
3β-25$(OH)D_3$	Light	649.4	340.3	28	40	45	11	15
d_6-3 β -25$(OH)D_3$ (IS)	Light	655.4	340.3	28	40	45	11	15
Vitamin D_3	Light	633.4	280.3	38	40	40	13	13
d_3-Vitamin D_3	Light	636.4	283.3	38	40	40	13	13
24,25$(OH)_2D_3$	Heavy	713.5	343.3	30	40	45	11	15
d_6-24,25$(OH)_2D_3$ (IS)	Heavy	719.5	343.3	30	40	45	11	15
3α-25$(OH)D_3$	Heavy	652.4	343.3	28	40	45	11	15
d_6-3α-25$(OH)D_3$ (IS)	Heavy	658.4	343.3	28	40	45	11	15
3β-25$(OH)D_3$	Heavy	652.4	343.3	28	40	45	11	15
d_6-3β -25$(OH)D_3$ (IS)	Heavy	658.4	343.3	28	40	45	11	15
Vitamin D_3	Heavy	636.4	280.3	38	40	40	13	13
d_3-Vitamin D_3	Heavy	639.4	283.3	38	40	40	13	13

Table S2: Concentration levels in ng mL^{-1}, precision and accuracy of calibration samples expressed as relative error (RE) and relative standard deviation (RSD; n=3) in %.

Quality control samples	**24,25(OH)$_2$D$_3$**					**3α-25(OH)D$_3$**					**3β-25(OH)D$_3$**					**Vitamin D$_3$**				
	Conc	Ligth tag		Heavy tag		Conc	Ligth tag		Heavy tag		Conc	Ligth tag		Heavy tag		Conc	Ligth tag		Heavy tag	
Calibration level	ng mL^{-1}	RE [%]	RSD [%]	RE [%]	RSD [%]	ng mL^{-1}	RE [%]	RSD [%]	RE [%]	RSD [%]	ng mL^{-1}	RE [%]	RSD [%]	RE [%]	RSD [%]	ng mL^{-1}	RE [%]	RSD [%]	RE [%]	RSD [%]
L1	0.1	0.1	2.8	3.2	2.9	0.1	1.3	0.9	1.6	2.7	1	10.4	2.1	12.7	2.8	0.35	1.0	1.0	0.2	0.6
L2	0.4	0.2	2.0	1.4	1.8	0.4	2.4	1.0	0.2	2.0	3	0.2	1.9	2.3	0.8	0.7	5.4	1.2	5.8	0.7
L3	1.7	0.6	2.2	0.2	0.7	1.7	1.1	3.3	1.4	0.5	10	5.7	3.0	5.5	0.3	2.7	1.7	1.2	2.1	0.9
L4	5	0.2	0.6	3.7	2.0	5	1.5	1.2	1.8	2.3	20	6.9	1.6	7.0	1.4	9.7	3.9	2.2	3.9	1.2
L5	10	2.1	2.5	2.2	1.1	10	1.4	0.7	2.4	0.8	40	0.4	0.9	0.0	1.8	19.7	2.2	1.7	1.3	1.7
L6	17.5	1.0	1.2	0.1	1.1	17.5	0.0	1.1	0.4	1.4	70	0.6	1.1	0.6	0.8	39.7	0.5	1.2	0.7	0.8
L7	25	0.1	0.7	0.0	0.8	25	0.2	1.8	0.2	0.1	100	1.3	1.6	1.5	0.8	69.7	0.9	2.5	0.5	0.2
